# Comparative Biodistribution Study of Baculoviral and Adenoviral Vector Vaccines against SARS-CoV-2

**DOI:** 10.4014/jmb.2308.08042

**Published:** 2023-10-11

**Authors:** Hyeon Dong Lee, Jungmin Chun, Sehyun Kim, Nowakowska Aleksandra, Chanyeong Lee, Doyoung Yoon, Hee-jung Lee, Young Bong Kim

**Affiliations:** 1 Department of Biomedical Science and Engineering, Konkuk University, Seoul 05029, Republic of Korea; 2 KR BioTech Co. Ltd., Seoul 05029, Republic of Korea

**Keywords:** Biodistribution, SARS-CoV-2, baculoviral vector, adenoviral vector, vaccine

## Abstract

Various types of vaccines have been developed against COVID-19, including vector vaccines. Among the COVID-19 vaccines, AstraZeneca’s chimpanzee adenoviral vaccine was the first to be commercialized. For viral vector vaccines, biodistribution studies are critical to vaccine safety, gene delivery, and efficacy. This study compared the biodistribution of the baculoviral vector vaccine (AcHERV-COVID19) and the adenoviral vector vaccine (Ad-COVID19). Both vaccines were administered intramuscularly to mice, and the distribution of the SARS-CoV-2 S gene in each tissue was evaluated for up to 30 days. After vaccination, serum and various tissue samples were collected from the mice at each time point, and IgG levels and DNA copy numbers were measured using an enzyme-linked immunosorbent assay and a quantitative real-time polymerase chain reaction. AcHERV-COVID19 and Ad-COVID19 distribution showed that the SARS-CoV-2 spike gene remained predominantly at the injection site in the mouse muscle. In kidney, liver, and spleen tissues, the AcHERV-COVID19 group showed about 2–4 times higher persistence of the SARS-CoV-2 spike gene than the Ad-COVID19 group. The distribution patterns of AcHERV-COVID19 and Ad-COVID19 within various organs highlight their contrasting biodistribution profiles, with AcHERV-COVID19 exhibiting a broader and prolonged presence in the body compared to Ad-COVID19. Understanding the biodistribution profile of AcHERV-COVID19 and Ad-COVID19 could help select viral vectors for future vaccine development.

## Introduction

Various vaccine platforms have been developed for COVID-19 global pandemic caused by severe acute respiratory syndrome coronavirus 2 (SARS-CoV-2). COVID-19 vaccines based on mRNA and adenoviral vectors are approved systems that induce antibody production by expressing the COVID-19 viral spike protein as an endogenous antigen [1]. Viral vectors used in vaccine development include adenoviruses, measles, influenza, poxvirus, parainfluenza, Sendai, and rabies viruses [2-4]. Although animal-derived viral vectors excel in efficiently entering human cells, they often encounter challenges related to antibody formation and toxicity [5]. To overcome the problems of viral vector vaccines, a DNA vaccine platform technology using baculoviral vector was developed previously [6, 7].

Baculovirus-based DNA vaccines can transduce target genes with high efficacy. Their replicative incompetence in mammalian cell systems contributes to their safety in vertebrates and humans, minimizing the risk of reversion and unintended vector gene expression [7]. Moreover, baculoviral vectors allow easy genetic manipulation of high-capacity genes.

In our previous study, we developed AcHERV-COVID19 using the baculovirus vector system and evaluated it in K18-hACE2 transgenic mice. The results demonstrated that the generated vaccine could potentially provide protection against Delta and Omicron BA1 variants [6]. Despite their superior efficacy, information on the biodistribution of baculovirus vaccines is limited, and an understanding of their biodistribution and safety profiles is needed. In this study, we aimed to investigate the biodistribution of the AcHERV-COVID19 vaccine and compare it with an adenoviral vector vaccine.

## Materials and Methods

### Cells

*Spodoptera frugiperda* 9 (Sf9; Invitrogen, USA) cells were propagated in Sf-900II medium (Invitrogen, USA). African green monkey kidney clone E6 cells (Vero E6, ATCC, USA) and 293T cells (ATCC, USA) were maintained in Dulbecco’s modified Eagle’s medium (Thermo Fisher Scientific, USA) at 37°C.

### Viral Vectors and Recombinant Viruses

Previously we reported the recombinant baculovirus, AcHERV-COVID19 [6]. As a baculoviral vector, the gene encoding the human endogenous retrovirus (HERV) protein was inserted downstream of the polyhedrin promoter into the pFastBacTM1 plasmid. The SARS-CoV-2 spike gene was inserted downstream of the cytomegalovirus (CMV) promoter. AcHERV-COVID19 was produced using the Bac-to-Bac Baculovirus Expression System (Thermo Fisher Scientific). Quantification of AcHERV-COVID19 was carried out using BacPAK qPCR Titration Kit (Takara, Japan). The quantified DNA copy number was converted to infectious forming unit (IFU) according to the manufacturer manual [8]. Ad-COVID19, an adenoviral recombinant virus, was produced in 293T cells by introducing a sequence encoding the spike gene into the adenoviral vector. The recombinant adenovirus was constructed using an Adeno-X Adenoviral System 3 kit (Takara) manual. The SARS-CoV-2 spike gene was amplified using forward (5'-GTAACTATAACGGTCAGCGTTTAAACTTAAGCTTGGT ACC-3') and reverse (5'-TTACCTCTTTCTCCTCAGGTGTAGTGCAGTTTCACG-3') primers from recombinant baculovirus, AcHERV-COVID19. Quantification of Ad-COVID19 was carried out using an Adeno-X qPCR Titration kit (Takara). The quantified DNA copy number was converted to IFU according to the Adeno-X Adenoviral System 3 kit manufacturer manual.

### Western blotting and Immunofluorescence

Vero E6 cells were infected with the recombinant virus at 30 multiplicities of infection (moi). Three days after infection, immunofluorescence analysis and western blotting were performed, as previously described, using a SARS-CoV-2 polyclonal antibody (Elabscience, USA) [6].

### Animal Experiments

Six-week-old female BALB/c mice were purchased from Orient Bio (Korea). The mice were supplied with food and water *ad libitum*. All animal experiments were approved by the Konkuk University Institutional Animal Care and Use Committee (IACUC approval no: KU22229-1) and conducted in strict compliance with the Guide for the Care and Use of Laboratory Animals of the National Institute of Health. Both vaccines were administered at a dose of 100 μl per mouse with 1 × 10^8^ IFU in hind leg. The viral titer of each vaccine was calculated to SARS-CoV-2 S copy number, which was 8.40 × 10^7^ copies/ml for AcHERV-COVID19 and 6.40 × 10^6^ copies/ml for Ad-COVID19.

### Blood and Tissue Collection

Following AcHERV-COVID19 or Ad-COVID19 administration, serum was assessed, and tissues (administration site, inguinal lymph nodes, ovaries, kidneys, spleens, livers, thymus glands, lungs, hearts, and brains) were collected after anesthesia. At each time point, blood and tissue samples were collected.

### Quantitative Real-Time Polymerase Chain Reaction (qPCR)

DNA was isolated from tissues and purified using an AccuPrep Genomic DNA Extraction Kit (Bioneer, Korea). Amplification of 200 ng of gDNA sample was performed under the following conditions: initial denaturation at 95°C for 30 s, followed by 40 cycles of denaturation at 95°C for 3 s, and annealing/extension at 60°C for 31 s. To use the DNA standard curve method, T-SARS-CoV-2S was prepared by introducing the spike gene of SARS-CoV-2 into a pGEM-T vector (Thermo Fisher Scientific). The concentration of T-SARS-CoV-2S relative to the DNA standard curve was determined using qPCR. The resulting DNA concentration was calculated as copies/200 ng of DNA.

The SARS-CoV-2 spike region was amplified using forward (5'-GTCTAA TCTCAAACCTTTTGAGAG-3') and reverse (5'-ACAGTTGCTGGTGCATGTAGAAGT-3') primers. The primer sequences used to amplify glyceraldehyde 3-phosphate dehydrogenase (*GAPDH*) were as follows: forward 5'-TCACCACCATGGAGAAGGC-'3, and reverse 5'-GCTAAGCAGTTGGTGGTGCA-3'.

### Enzyme-Linked Immunosorbent assay (ELISA)

Antibodies specific to the SARS-CoV-2 spike protein were detected using ELISA. 96-well plate was coated with 50 ng/well of the SARS-CoV-2 spike receptor binding protein produced in our laboratory using the Sf9 cell expression system. 1/100 dilution of mouse serum sample (0.06 ml/well) was loaded and incubated for 3 h at the temperature of 25°C. The plates were washed and treated with peroxidase-conjugated goat anti-mouse IgG antibody (1:10000, Abcam, UK). Absorbance was measured at 450 nm using an Epoch microplate reader (BioTek Instruments, USA). Each experiment was repeated three times.

## Results

### Delivery Efficiency and Expression of the SARS-CoV-2 Spike Protein

**Fig. 1:**
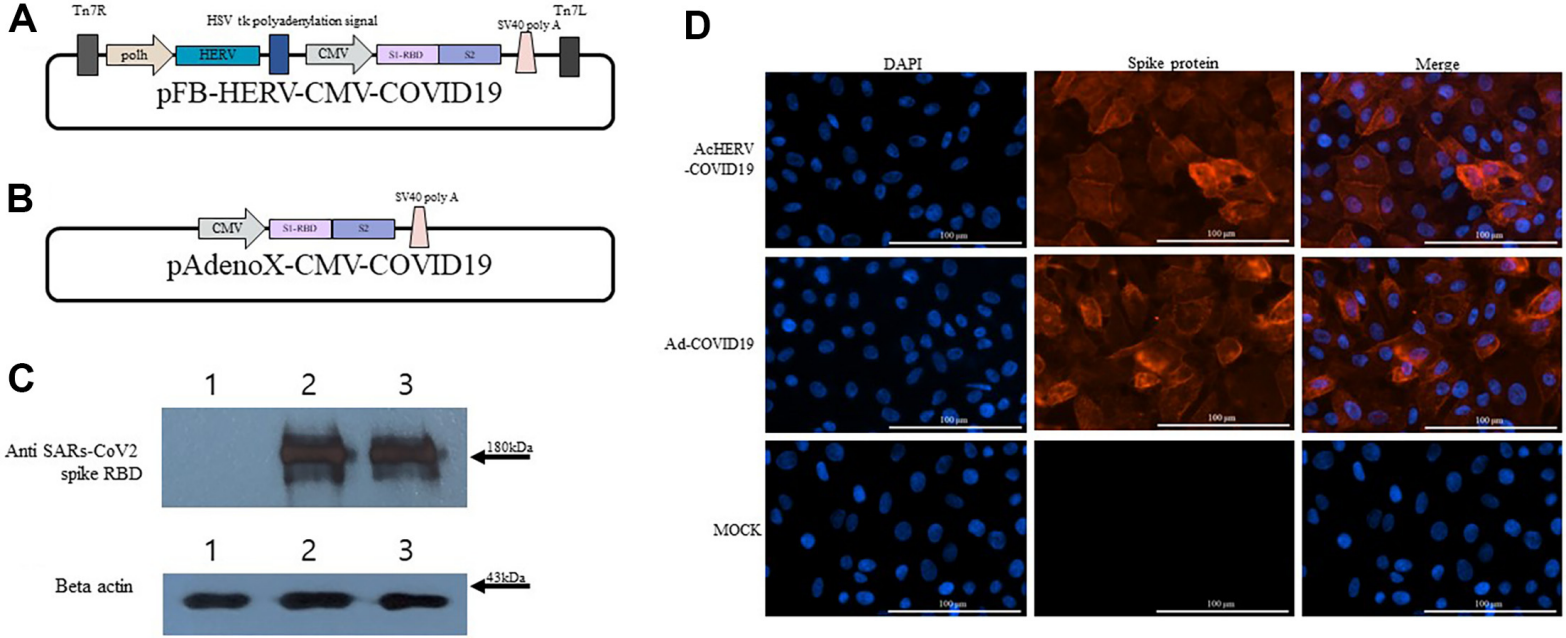
Schematic diagram of two recombinant plasmids and SARS-CoV-2 spike protein expression. (**A**) The pFB-HERV-CMV-COVID19 plasmid was constructed for the baculoviral vaccine (AcHERV-COVID19). (**B**) The pAdenoXCMV-COVID19 plasmid was constructed for the adenoviral vaccine (Ad-COVID19). (**C**) Expression of the SARS-CoV-2 spike protein by western blotting. Vero E6 cells were infected with AcHERV-COVID19 or Ad-COVID19. Lane 1: Vero E6 cell lysates (mock infection); Lane 2: AcHERV-COVID19-infected cells; Lane 3: Ad-COVID19-infected cells. (**D**) Expression of the SARS-CoV-2 spike protein using an immunofluorescence assay.

In this study, the baculoviral vaccine AcHERV-COVID19 was designed based on an existing vaccine used in previous research. This vaccine delivers the spike gene of the SARS-CoV-2, aiming to express the spike antigen after intramuscular injection. Similarly, the adenovirus vaccine was constructed based on an adenovirus vector, incorporating the sequence of the spike gene of the same variant of SARS-CoV-2, leading to the creation of Ad -COVID19 (Fig. 1A and 1B).

The transduced spike gene was expressed at a similar level in the two viral vector systems (Fig. 1C). Immunofluorescence analysis also showed similar fluorescence intensities in the two groups, and similar levels of protein expression in the two recombinant viruses were observed. Therefore, no difference in the expression level of the spike protein when cells were infected with the same amount (30 moi) of AcHERV-COVID19 and Ad-COVID19 (Fig. 1D) was observed.

### Standardization for SARS-CoV-2 S Gene Quantification

Quantification of the SARS-CoV-2 S gene was performed using real-time qPCR with DNA extracted from each organ. A standard curve for the gene was constructed using the pGEM-T vector and the DNA concentration in each organ was calculated using this standard curve. The validation of the qPCR method for each tissue is shown in Table 1. The standard curve of DNA extracted from the muscles of immunized mice showed reliable data, with a slope of -3.354, a correlation coefficient (R^2^) of 0.999, an amplification efficiency of 98.70%, and a coefficient of variation (CV) of 2.61%. The standard curve for organs other than muscle had a slope from -3.20 to -3.46, an R^2^ from 0.997 to 1, and an amplification efficiency from 94.54% to 105.54%; the CV ranged from 0.7% to 3.5%. The distribution of the SARS-CoV-2 S gene in the viral vectored vaccines was quantified using the copy number of the specific SARS-CoV-2 S gene for DNA extracted from each organ and blood: inguinal lymph nodes, ovary, spleen, kidney, liver, thymus, lung, heart, brain, and blood (Table 1).

### Serum IgG of the Two Vaccines Following a Single Intramuscular Injection

**Fig. 2:**
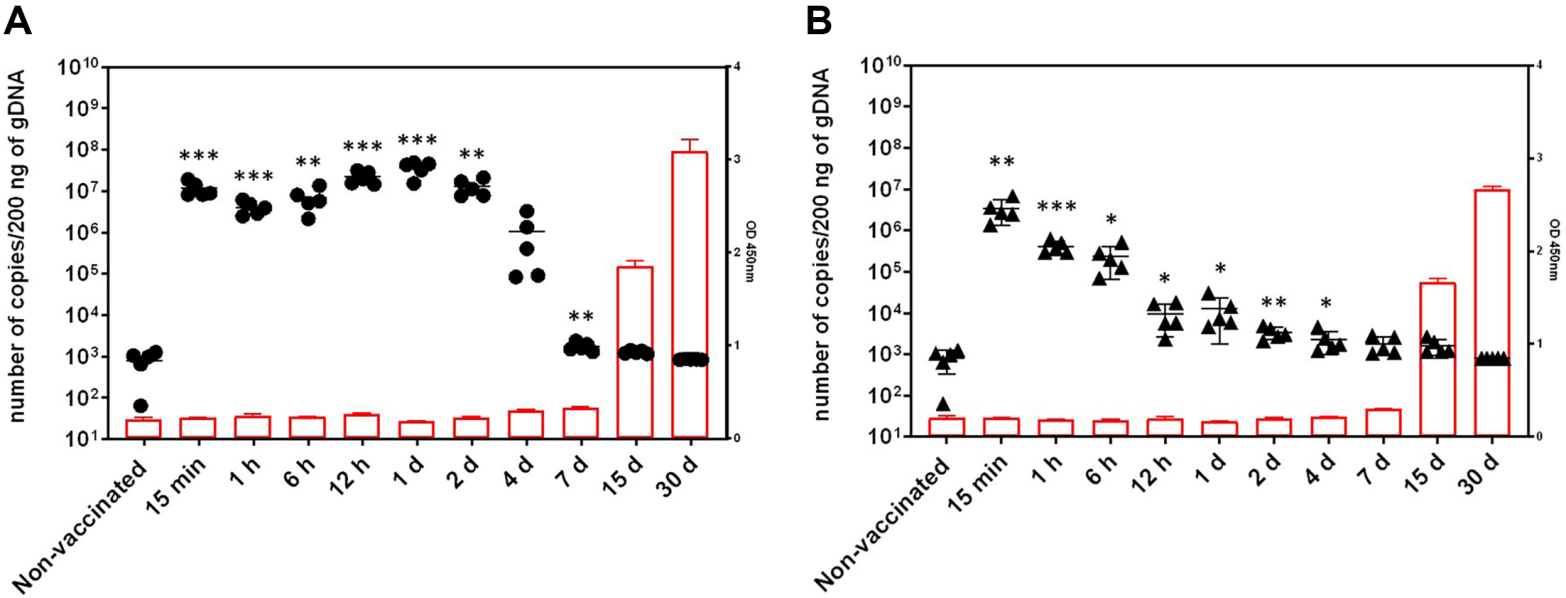
Time-dependent SARS-CoV-2 S gene persistence and IgG levels. (**A**) Residual amount of target DNA from AcHERV-COVID19 and IgG. (**B**) Residual amount of target DNA of Ad-COVID19 and IgG. The number of copies/200 ng of gDNA was measured using quantitative PCR from each tissue at various time points after immunization with both vaccines. Results are expressed as the mean ± SD (standard deviation). *P* values were calculated using two-tailed unpaired Student’s *t*-test (*p < 0.05, **p < 0.01, ***p < 0.001 for comparisons between non-immunized group; *n* = 5).

To evaluate the immunogenicity of SARS-CoV-2 S protein-specific total IgG levels, the serum of the tested group immunized with Ad-COVID19 and AcHERV-COVID19 was collected (Fig. 2). The immune response induced by the two vaccines was determined by ELISA, specifically targeting IgG antibodies against the SARS-CoV-2 spike protein. In the AcHERV-COVID19 immunized group, IgG increased 6.78 times (6.66–6.92) at 15 days after immunization and 11.28 times (10.95–11.60) at 30 days after immunization compared to that of 15 min after immunization (Fig. 2A). In the Ad-COVID19 immunized group, IgG increased 6.08 times (5.97–6.18) at the 15th day after immunization and 9.74 times (9.68–9.81) at the 30th day after immunization compared to that of 15 min after immunization (Student’s *t*-test; *p* < 0.0001 comparisons between non-immunized group) (Fig. 2B). Increases in IgG and antibody levels in both groups were similar, and no differences were observed between the two groups.

### Persistence of the Two Vaccines at the Injection Site Following a Single Intramuscular Injection

After intramuscular inoculation of mice with the AcHERV-COVID19 or Ad-COVID19 vaccines, the biodistribution profile in the muscle of the inoculation site (hind leg) was examined over time. Both vaccines were administered at a dose of 1 × 10^8^ IFU/100 μl to each mouse.

The SARS-CoV-2 S gene was quantitatively detected in all tissues of the injected mice. The SARS-CoV-2 S gene retention in the muscles of mice inoculated with AcHERV-COVID19 was 1.18 × 10^7^, 4.06 × 10^6^, 6.96 × 10^6^, and 2.20 × 10^7^ copies/200 ng DNA after 15 min, 1 h, 6 h, and 12 h, respectively. The remaining amount (copies/200 ng DNA) was 3.71 × 10^7^, 1.30 × 10^7^, 1.04 × 10^6^, 949, 473, and 50 copies/200 ng DNA at 1, 2, 4, 7, 15, and 30-days post injection (dpi), respectively. Over time, the amount of DNA remaining in the muscle seemed to be maintained until day 2, and then it rapidly decreased from day 4. Very low amounts were detected on days 7 and 15, and no DNA was observed on day 30 after inoculation (Fig. 2A).

For Ad-COVID19, the remaining amount (copies/200ng DNA) was 3.41 × 10^6^ at 15 min after inoculation, 4.14 × 10^5^ at 1 h, 2.37 × 10^5^ at 6 h, 8870 at 12 h, 1.17 × 10^5^ at 1 dpi, 2557 at 2 dpi, 1481 at 4 dpi, 985 at 7 dpi, 821 at 15 dpi, and undetectable at 30 dpi. Ad-COVID19 showed an almost 4000-fold decrease in initial residual DNA (15 min) compared to the dose, with a lower level (approximately 8-fold lower than 12 h) between 12 h and 15 dpi. Complete ablation of the SARS-CoV-2 S gene was observed on day 30 (Fig. 2B).

The SARS-CoV-2 S gene in the muscle, the site of administration, gradually decreased over time for AcHERV-COVID19 and Ad-COVID19 vaccines after administration. AcHERV-COVID19 rapidly decreased from 4 dpi to 7 dpi after administration and then from 7 dpi, it gradually decreased and almost disappeared at 30 dpi.

Ad-COVID19 temporarily increased at 12 h and then rapidly decreased. SARS-CoV-2 S gene remained for a longer period in AcHERV-COVID19 than in Ad-COVID19. However, the gene was not retained in either vaccine at 30 dpi until the end of the experiment.

### Biodistribution of the Two Vaccines Following a Single Intramuscular Injection

**Fig. 3:**
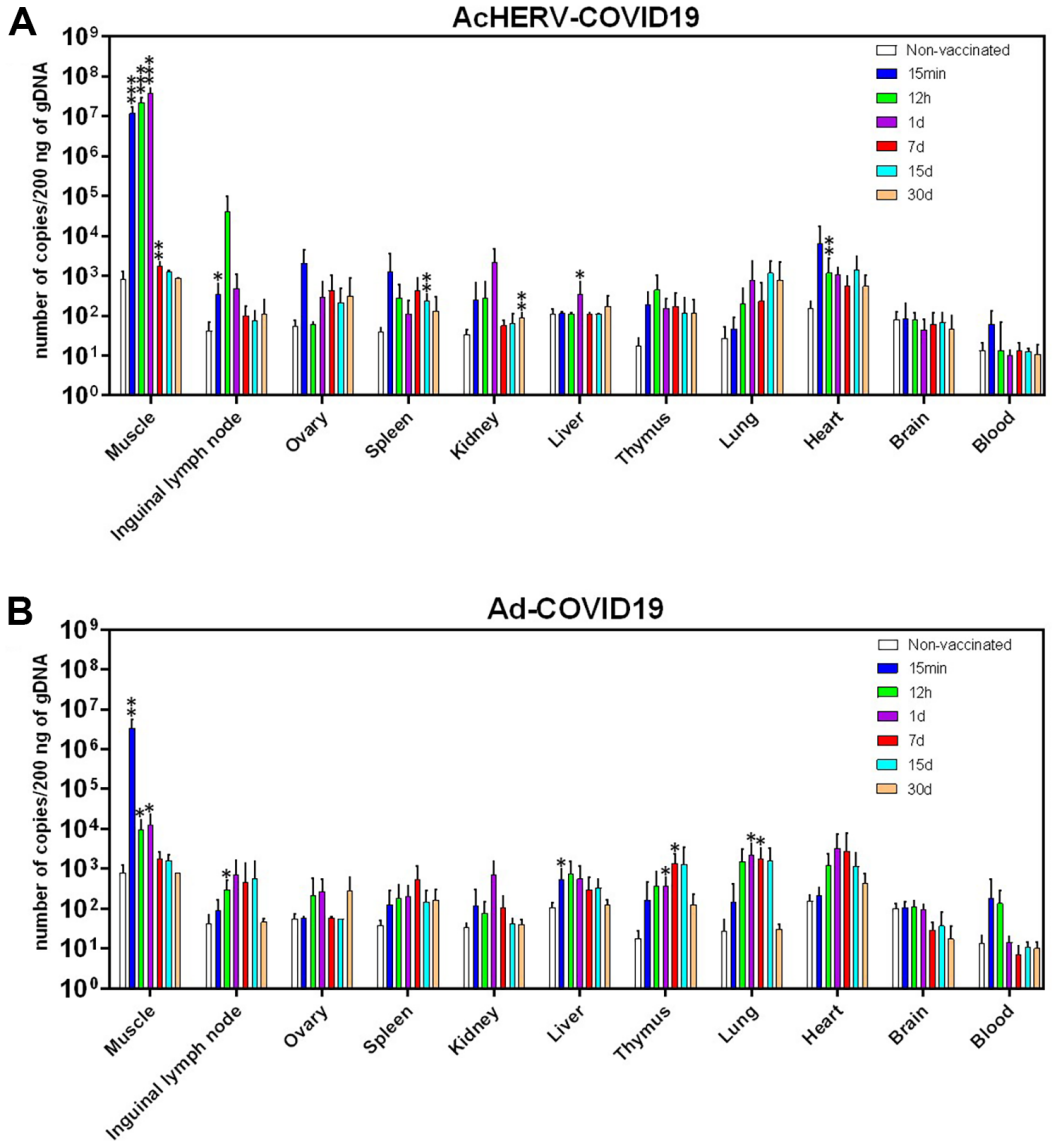
Biodistribution of AcHERV-COVID19 and Ad-COVID19 vaccines following a single intramuscular injection. After intramuscular immunization of the AcHERV-COVID19 and Ad-COVID19 the copy number of the SARS-CoV-2 S gene (copies/200 ng) was measured in gDNA extracted from various tissues. *P* values were calculated using two-tailed unpaired Student’s *t*-test (*p < 0.05, **p < 0.01, ***p < 0.001 for comparisons between non-immunized group; *n* = 5).

Following the AcHERV-COVID19 or Ad-COVID19 vaccine injection into the hind legs of mice, the biodistribution profile in various tissues was investigated over time. For both vaccines, very high levels of DNA copy number (1.5 × 10^3^ – 5.0 × 10^7^ copies/200 ng DNA) were observed in the muscle at the injection site between 15 min and 1 dpi (Fig. 3).

When AcHERV-COVID19 was inoculated, the SARS-CoV-2 S gene was observed at around 1.4 × 10^3^ – 9.5 × 10^3^ copies/200 ng DNA in most tissues except the liver, brain, and blood from the first 15 min to 1 dpi (Fig. 3A). Afterwards, the DNA levels decreased from 7 dpi and were below 243 copies/200 ng DNA at 30 dpi at the end of the experiment. DNA in the liver tissue was not detectable before 1 dpi but was detected after 1 dpi or more. Fifteen minutes after injection, the SARS-CoV-2 S gene was detected in the blood samples, but little DNA was detected in the brain tissue. Specifically, the DNA copy number gradually increased in the lungs, peaking at 15 dpi.

For Ad-COVID19, DNA copy numbers were low immediately after inoculation (15 min) in most tissues except muscle, subsequently showing a gradual increase over time. In the brain tissue, low levels of DNA copy numbers were observed at 1 and 7 dpi and not observed again after 15 days. For AcHERV-COVID19, the SARS-CoV-2 S gene was detected in the blood samples 15 min after injection, whereas no DNA was detected at later time points (Fig. 3B).

Based on biodistribution profiles, the AcHERV-COVID19 group had a high copy number of SARS-CoV-2 S DNA until 12 h after inoculation, but the Ad-COVID19 group had a relatively high number of DNA copies after 1 day. This implies that AcHERV-COVID19 rapidly transfers genes not only to the site of inoculation but also to various organs. Furthermore, the median DNA copy number in tissues other than muscle was 1354 copies/200 ng DNA for AcHERV-COVID19 and 350 copies/200 ng DNA for Ad-COVID19, indicating that AcHERV-COVID19 was more persistent.

## Discussion

Biodistribution analysis is a crucial step in evaluating vaccine safety and efficacy [9]. Gene therapies and vaccines usually require biodistribution assessments to ensure that the vector reaches its intended target cells and does not harm other cells or organs. Various studies have evaluated the viral vector-based vaccine biodistribution and identified potentially toxic target organs. Biodistribution of vaccines involves the administration and delivery of therapeutic agents, such as antibodies and antigens, to individuals through various routes, such as intramuscular injection and oral administration [10-13]. Biodistribution plays an important role in assessing a vaccine’s immunogenicity and safety (toxicity) because it ensures that the vaccine efficiently reaches the target cells or organs. In addition, biodistribution provides data for immunization strategies by monitoring the route of administration, appropriate dosage, and post-vaccination side effects [12, 14]. Therefore, the clear identification of the biodistribution of vaccines plays an essential role in the prevention and treatment of infectious diseases.

Most studies have administered viruses via the intravenous route, but some studies have explored the distribution of viral vectors via intramuscular, intranasal, and intratracheal routes [15-18]. One study evaluated the non-clinical immunogenicity, biodistribution, and toxicity of a chimpanzee adenovirus-based COVID-19 vaccine in mice and rhesus monkeys [19]. Another study evaluated the differential biodistribution of adenovirus-vectorized vaccines after intranasal and intratracheal delivery and found that vaccine biodistribution correlated with immunogenicity and protection [20]. Therefore, the biodistribution of a vaccine is important for immunogenicity evaluation.

Over the past 20 years, baculoviruses have been used as a research tool for transient transgene expression [21]. Although not yet directly used as a gene therapy vector in a clinical setting, numerous preclinical studies have suggested the promising potential of baculoviruses as delivery vectors for a variety of therapeutic applications, including vaccines, tissue engineering, and cancer treatment. Therefore, we are interested in vaccines using baculoviral vectors, which has led to the development of baculovirus-based DNA vaccines [6].

AcHERV-COVID19 can be distributed from the injection site to other parts of the body. Here, the biodistribution of the AcHERV-COVID19 vaccine was evaluated according to the World Health Organization (WHO) guidelines for the non-clinical evaluation of vaccines (WHO Technical Report, Series No. 927, 2005; EMA CHMP/VWP/141697/2009) [22].

Biodistribution assessment showed that after a single intramuscular injection, AcHERV-COVID19 was predominantly present in the muscle at the injection site in mice, and the viral DNA (SARS-CoV-2 S) copy number gradually decreased over time. It has been reported a long time ago that baculovirus is replication incompetent in mammalian cells. We confirmed its replication incompetency in 293T cell infected with baculovirus and identified it through RNA-seq analysis [23]. However, Fig. 2 showed the viral qPCR data looks like increasing in the early stage. In this study, we purified gDNA from tissue using a gDNA purification kit and performed qPCR. It is thought that the purification efficiency of viral DNA was reduced in the early stages of baculovirus injection into mice because viral DNA was purified from muscle at a stage when the virus had not entered the cells. On the other hand, after the virus entered the cells (at 12 h), DNA copy seemed to increase as the purification efficiency of viral DNA increased. Therefore, the increase in baculovirus DNA copy number at the beginning of virus injection is thought to be due to differences in DNA purification efficiency.

The Food and Drug Administration (FDA) and European Medicines Agency (EMA) suggest methods for in vivo biodistribution studies. Although vector detection in samples from the in vivo experiments in the study has not been described in the EMA documentation, FDA documentation provides sufficient details [24, 25]. Additionally, the most recent pharmacokinetic studies on DNA vaccines have employed qPCR, which is described as the most common and reliable method for assessing plasmid levels in biodistribution studies among the available nucleic acid detection techniques [26-28].

The detection limit of our qPCR assay was 10 copies/200 ng of gDNA in all tested tissues, which satisfies the regulatory agency guidelines. With a detection limit of 50 copies/200 ng DNA and CV values of < 4.0% (Table 1), our qPCR assays showed high sensitivity and reproducibility.

FDA guidelines require that integration studies be performed when the copy numbers of DNA vaccines are >30,000/μg of host DNA 90 days after administration [22, 24]. Although our AcHERV-COVID19 vaccine persisted longer than the Ad-COVID19 vaccine at the injection site, the levels of the SARS-CoV-2 S gene were already < 254 copies/200ng of gDNA 30 days after intramuscular administration.

Notably, multiple examinations at various time points after administration via the intramuscular route revealed systemic tissue distribution and prolonged retention of the AcHERV-COVID19 vaccine (Fig. 3). AcHERV-COVID19 may be distributed to organs other than muscle via the lymphatic network after intramuscular injection. reported that baculoviruses may spread to other organs via the lymphatic network after administration via different routes (intramuscular, intraperitoneal, and intracerebroventricular) in rats [29].

In conclusion, this study is the first to investigate the biodistribution of a baculoviral DNA vaccine (AcHERV-COVID19) against SARS-CoV-2. We demonstrated that the biodistribution profile of the AcHERV-COVID19 vaccine was different from that of Ad-COVID19. This may contribute to knowledge regarding the in vivo administration of baculovirus vectors and provide non-clinical data to understand the pharmacodynamics and safety of this vaccine system. In addition, this study may contribute to clinical trials of new vaccine systems using baculovirus-based genetic vaccines.

## Figures and Tables

**Table 1:** Validation of the qPCR method in each tissue.

Tissue type	Slope	Correlation coefficients (R^2^)	Amplification efficiency (%)	CT value (mean ± SD)	CV (%)^a^
Muscle	-3.35	0.999	98.70	13.76 ± 0.36	2.61
Inguinal lymph node	-3.22	0.999	104.56	13.41 ± 0.39	2.91
Ovary	-3.25	1.000	102.91	14.48 ± 0.28	1.93
Spleen	-3.45	0.999	94.83	14.35 ± 0.50	3.50
Kidney	-3.25	0.998	103.17	14.86 ± 0.25	1.67
Liver	-3.23	0.999	104.03	15.76 ± 0.26	1.62
Thymus	-3.46	0.996	94.54	14.73 ± 0.24	1.76
Lung	-3.39	0.997	97.47	13.55 ± 0.16	1.20
Heart	-3.26	0.999	102.58	13.90 ± 0.10	0.70
Brain	-3.20	1.000	105.54	14.29 ± 0.43	3.04
Blood	-3.39	0.999	97.33	13.88 ± 0.33	2.38

Ten-fold serial dilutions of T-SARS-CoV-2_S, ranging from 1 × 10^1^ to 1 × 10^8^ copies/μl, were used to construct the standard curves in solutions containing 200 ng of gDNA from each tissue.

^a^The amplification of *GAPDH* in solutions containing 200 ng of gDNA from each tissue (*n* = 6).

## References

[1] Kyriakidis NC, Lopez-Cortes A, Gonzalez EV, Grimaldos AB, Prado EO (2021). SARS-CoV-2 vaccines strategies: a comprehensive review of phase 3 candidates. NPJ Vaccines.

[2] Travieso T, Li J, Mahesh S, Mello J, Blasi M (2022). The use of viral vectors in vaccine development. NPJ Vaccines.

[3] Deng S, Liang H, Chen P, Li Y, Li Z, Fan S (2022). Viral vector vaccine development and application during the COVID-19 pandemic. Microorganisms.

[4] Lundstrom K (2021). Viral vectors for COVID-19 vaccine development. Viruses.

[5] McCann N, O'Connor D, Lambe T, Pollard AJ (2022). Viral vector vaccines. Curr. Opin. Immunol..

[6] Jang Y, Cho H, Chun J, Park K, Nowakowska A, Kim J (2023). Baculoviral COVID-19 Delta DNA vaccine cross-protects against SARS-CoV2 variants in K18-ACE2 transgenic mice. Vaccine.

[7] Cho H, Jang Y, Park KH, Choi H, Nowakowska A, Lee HJ (2021). Human endogenous retrovirus-enveloped baculoviral DNA vaccines against MERS-CoV and SARS-CoV2. NPJ. Vaccines.

[8] Gwon YD, Kim S, Cho Y, Heo Y, Cho H, Park K (2016). Immunogenicity of virus like particle forming baculoviral DNA vaccine against pandemic influenza H1N1. PLoS One.

[9] Naasani I (2022). Establishing the pharmacokinetics of genetic vaccines is essential for maximising their safety and efficacy. Clin. Pharmacokinet..

[10] Shimada M, Wang H, Ichino M, Ura T, Mizuki N, Okuda K (2022). Biodistribution and immunity of adenovirus 5/35 and modified vaccinia Ankara vector vaccines against human immunodeficiency virus 1 clade C. Gene Ther..

[11] Li LH, Liesenborghs L, Wang L, Lox M, Yakass MB, Jansen S (2022). Biodistribution and environmental safety of a liveattenuated YF17D-vectored SARS-CoV-2 vaccine candidate. Mol. Ther. Methods Clin. Dev..

[12] Stebbings R, Armour G, Pettis V, Goodman J (2022). AZD1222 (ChAdOx1 nCov-19): A single-dose biodistribution study in mice. Vaccine.

[13] Sheets RL, Stein J, Bailer RT, Koup RA, Andrews C, Nason M (2008). Biodistribution and toxicological safety of adenovirus type 5 and type 35 vectored vaccines against human immunodeficiency virus-1 (HIV-1), Ebola, or Marburg are similar despite differing adenovirus serotype vector, manufacturer's construct, or gene inserts. J. Immunotoxicol..

[14] Yang R, Deng Y, Huang B, Huang L, Lin A, Li Y (2021). A core-shell structured COVID-19 mRNA vaccine with favorable biodistribution pattern and promising immunity. Signal Transduct. Target. Ther..

[15] Driedonks T, Jiang L, Carlson B, Han Z, Liu G, Queen SE (2022). Pharmacokinetics and biodistribution of extracellular vesicles administered intravenously and intranasally to *Macaca nemestrina*. J. Extracell. Biol..

[16] Pan D, Gunther R, Duan W, Wendell S, Kaemmerer W, Kafri T (2002). Biodistribution and toxicity studies of VSVG-pseudotyped lentiviral vector after intravenous administration in mice with the observation of in vivo transduction of bone marrow. Mol. Ther..

[17] Kang M, Jordan V, Blenkiron C, Chamley LW (2021). Biodistribution of extracellular vesicles following administration into animals: a systematic review. J. Extracell. Vesicles.

[18] Wang L, Rao Y, Liu X, Sun L, Gong J, Zhang H (2021). Administration route governs the therapeutic efficacy, biodistribution and macrophage targeting of anti-inflammatory nanoparticles in the lung. J. Nanobiotechnology.

[19] Dai X, Zhao W, Tong X, Liu W, Zeng X, Duan X (2022). Non-clinical immunogenicity, biodistribution and toxicology evaluation of a chimpanzee adenovirus-based COVID-19 vaccine in rat and rhesus macaque. Arch. Toxicol..

[20] Jeyanathan V, Afkhami S, D'Agostino MR, Zganiacz A, Feng X, Miller MS (2022). Differential biodistribution of adenoviralvectored vaccine following intranasal and endotracheal deliveries leads to different immune outcomes. Front. Immunol..

[21] Condreay JP, Witherspoon SM, Clay WC, Kost TA (1999). Transient and stable gene expression in mammalian cells transduced with a recombinant baculovirus vector. Proc. Natl. Acad. Sci. USA.

[22] (2005). Guidelines for assuring the quality and nonclinical safety evaluation of DNA vaccines.

[23] Shin HY, Choi H, Kim N, Park N, Kim H, Kim J (2020). Unraveling the genome-wide impact of recombinant baculovirus infection in mammalian cells for gene delivery. Genes (Basel).

[24] Guidance for Industry: considerations for plasmid DNA vaccines for infectious disease indications.

[25] Cho HJ, Lee S, Im S, Kim MG, Lee J, Lee HJ (2012). Preclinical pharmacokinetics and biodistribution of human papillomavirus DNA vaccine delivered in human endogenous retrovirus envelope-coated baculovirus vector. Pharm. Res..

[26] Liu C, Fan M, Xu Q, Li Y (2008). Biodistribution and expression of targeted fusion anti-caries DNA vaccine pGJA-P/VAX in mice. J. Gene Med..

[27] Zhou QH, Wu C, Manickam DS, Oupicky D (2009). Evaluation of pharmacokinetics of bioreducible gene delivery vectors by real-time PCR. Pharm. Res..

[28] Fu J, Li D, Xia S, Song H, Dong Z, Chen F (2009). Absolute quantification of plasmid DNA by real-time PCR with genomic DNA as external standard and its application to a biodistribution study of an HIV DNA vaccine. Anal. Sci..

[29] Raty JK, Liimatainen T, Huhtala T, Kaikkonen MU, Airenne KJ, Hakumaki JM (2007). SPECT/CT imaging of baculovirus biodistribution in rat. Gene Ther..

